# P-1454. mRNA-Based Respiratory Syncytial Virus Vaccine mRNA-1345 Induces Robust, Polyfunctional, and Durable CD4+ and CD8+ T Cell Responses with Similar Features in Adults Across Age Groups

**DOI:** 10.1093/ofid/ofaf695.1640

**Published:** 2026-01-11

**Authors:** Yanbo Sun, Emily Marcisak, Daniel Makrinos, Jenna Landy, Shannon McGrath, Hsiaohsuan Kuo, Maria Cavallaro, Christopher Wu, Adam Essene, Haining Lin, Wen-Han Yu, Anthony DiPiazza, Jaap Oostendorp, Robert Paris

**Affiliations:** Moderna, Inc., Cambridge, Massachusetts; Moderna, Inc., Cambridge, Massachusetts; Moderna, Inc., Cambridge, Massachusetts; Moderna, Inc., Cambridge, Massachusetts; Moderna, Inc., Cambridge, Massachusetts; Moderna, Inc., Cambridge, Massachusetts; Moderna, Inc., Cambridge, Massachusetts; Moderna, Inc., Cambridge, Massachusetts; Moderna, Inc., Cambridge, Massachusetts; Moderna, Inc., Cambridge, Massachusetts; Moderna, Inc., Cambridge, Massachusetts; Moderna, Inc., Cambridge, Massachusetts; Moderna, Inc, Cambridge, MA; Moderna, Inc., Cambridge, Massachusetts

## Abstract

**Background:**

Respiratory syncytial virus (RSV) remains a major cause of morbidity and mortality, particularly among infants, the elderly, and individuals with high-risk conditions associated with severe outcomes. While neutralizing antibodies play a key role in protecting against initial infection and limiting viral replication, T cells are increasingly recognized as a critical component of the immune response to mitigate disease severity. Given the age-associated decline in T-cell function, evaluating T-cell responses to the mRNA-based RSV vaccine mRNA-1345 across age groups is important.

**Methods:**

In this clinical study (NCT05397223), healthy adults 18 to 75 years of age received 1 dose of mRNA-1345 (50 µg). Participant T-cell responses were evaluated by intracellular cytokine staining, activation-induced marker, and Meso Scale Discovery assays at several time points, up to 24 months after vaccination. Additionally, prefusion F (preF)-specific CD4^+^ and CD8^+^ T cells were sorted from peripheral blood mononuclear cells at baseline and 2 weeks after vaccination for single cell multiomic analysis.

**Results:**

mRNA-1345 induced robust CD4⁺ T-cell responses in both younger and older adults, with responses sustained at 24 months postvaccination. Vaccination with mRNA-1345 also elicited an increase in CD8⁺ T-cell frequencies. The CD4⁺ T-cell response was strongly Th1-polarized, characterized by elevated IFN-γ, IL-2, and TNF-α production, with minimal Th2 cytokine expression, indicating a favorable immune profile for viral control. Both CD4⁺ and CD8⁺ T cells exhibited increased polyfunctionality after vaccination and was consistent across age groups. Single-cell multiomic analysis revealed comparable phenotypic composition and transcriptional profiles of global and preF-specific T cells between younger and older adults. Additionally, T-cell receptor repertoire analysis demonstrated clonal overlap in vaccine-induced CD4⁺ and CD8⁺ T cells across age groups, supporting a shared clonal architecture of the vaccine-elicited response.

**Conclusion:**

Our results provide strong evidence that mRNA-1345 elicits durable and polyfunctional T-cell immunity in adults across age groups, with the potential to confer long-term protection against RSV.

**Disclosures:**

Yanbo Sun, PhD, Moderna, Inc.: Employee|Moderna, Inc.: Stocks/Bonds (Public Company) Emily Marcisak, MS, Moderna, Inc.: Employee|Moderna, Inc.: Stocks/Bonds (Public Company) Daniel Makrinos, MS, Moderna, Inc.: Employee|Moderna, Inc.: Stocks/Bonds (Public Company) Jenna Landy, BS, Moderna, Inc.: Employee|Moderna, Inc.: Stocks/Bonds (Public Company) Shannon McGrath, MS, Moderna, Inc.: Employee|Moderna, Inc.: Stocks/Bonds (Public Company) Hsiaohsuan Kuo, PhD, Moderna, Inc.: Employee|Moderna, Inc.: Stocks/Bonds (Public Company) Maria Cavallaro, BS, Moderna, Inc.: Employee|Moderna, Inc.: Stocks/Bonds (Public Company) Christopher Wu, MSc, Moderna, Inc.: Employee|Moderna, Inc.: Stocks/Bonds (Public Company) Adam Essene, MS, Moderna, Inc.: Employee|Moderna, Inc.: Stocks/Bonds (Public Company) Haining Lin, PhD, Moderna, Inc.: Employee|Moderna, Inc.: Stocks/Bonds (Public Company) Wen-Han Yu, PhD, Moderna, Inc.: Employee|Moderna, Inc.: Stocks/Bonds (Public Company) Anthony DiPiazza, PhD, Moderna, Inc.: Employee|Moderna, Inc.: Stocks/Bonds (Public Company) Jaap Oostendorp, PhD, Moderna, Inc.: Employee|Moderna, Inc.: Stocks/Bonds (Public Company) Robert Paris, MD, Moderna, Inc.: Employee|Moderna, Inc.: Stocks/Bonds (Public Company)

